# Acute myocarditis secondary to human metapneumovirus: a case series and mini-review

**DOI:** 10.21542/gcsp.2024.52

**Published:** 2024-12-31

**Authors:** Hussam Al Hennawi, Aamna Khan, Alexander Shpilman, Jennifer A. Mazzoni

**Affiliations:** 1 Department of Internal Medicine, Jefferson Abington Hospital Abington PA; 2 Department of Cardiology, Thomas Jefferson University Hospital Philadelphia PA

## Abstract

Among viral myocarditis, human metapneumovirus (hMPV) is a rare causative agent with associated cardiac complications. This report provides insight into the disease progression and sheds light on reported cases from the literature. We present the clinical courses of two patients, aged 68 and 58, who developed myocarditis secondary to hMPV infection. Despite the typical severity associated with viral myocarditis in older populations, both patients experienced significant improvement in their condition and ultimately survived. This report highlights the necessity of recognizing hMPV as a potential cause of myocarditis, particularly in older adults presenting with simultaneous respiratory and cardiac symptoms. The recovery and survival of these patients underscore the critical need for awareness and timely treatment strategies to manage this potentially severe condition.

## Introduction

Human metapneumovirus (hMPV), first identified in 2001, is a significant respiratory pathogen predominantly recognized for causing lower and upper respiratory tract infections. While it is commonly associated with illnesses such as bronchiolitis, pneumonia, and exacerbations of chronic obstructive pulmonary disease, hMPV has also been implicated in less frequent but severe extrapulmonary manifestations, including myocarditis. Myocarditis, an inflammation of the heart muscle, can result in a range of clinical outcomes from mild symptoms to severe heart failure and can lead to significant morbidity and mortality, particularly in older adults^1–3^.

In this case series, we present the clinical courses of two patients, aged 68 and 58, who developed myocarditis secondary to hMPV infection. Despite the typical severity associated with viral myocarditis in older populations, both patients experienced significant improvement in their condition and ultimately survived. These cases highlight the potential for favorable outcomes even in older adults with hMPV-associated myocarditis when appropriate and timely medical interventions are administered. Detailed descriptions of their clinical presentations, diagnostic evaluations, therapeutic approaches, and subsequent recovery provide valuable insights into managing this condition.

This case series underscores the importance of considering hMPV as a potential etiological agent in myocarditis, especially in older adults presenting with concurrent respiratory and cardiac symptoms. The survival and improvement seen in these patients emphasize the need for awareness and prompt treatment strategies to manage this potentially severe condition.

## Patient 1

A 68-year-old male presented to the emergency department in May with progressive dyspnea on exertion, lower extremity edema, and fatigue over a few weeks before arrival. His past medical history is limited to hypertension and retinal detachment. He reported visiting his primary care provider two months earlier for shortness of and non-productive cough and post-nasal drip, for which he was treated with steroids with partial improvement of symptoms. He takes no medications.

Upon arrival to the emergency department, he was noted to be volume overloaded, hypotensive in atrial flutter with rapid ventricular repones, and in respiratory distress. He had a temperature of 98.2 F, heart rate of 134 beats per minute, blood pressure of 96/65 mm Hg, and respiratory rate of 24 breaths per minute. An electrocardiogram (EKG) showed atrial flutter with 2:1 conduction. Blood work revealed a white blood count of 11.5, hemoglobin of 13.5, AST 476, ALT 575, creatinine 1.52, high sensitivity troponin T 79, which trended to 93, and lactate of 3.3. He was admitted to the intensive care unit and was initially managed with BiPAP.

**Figure 1.**
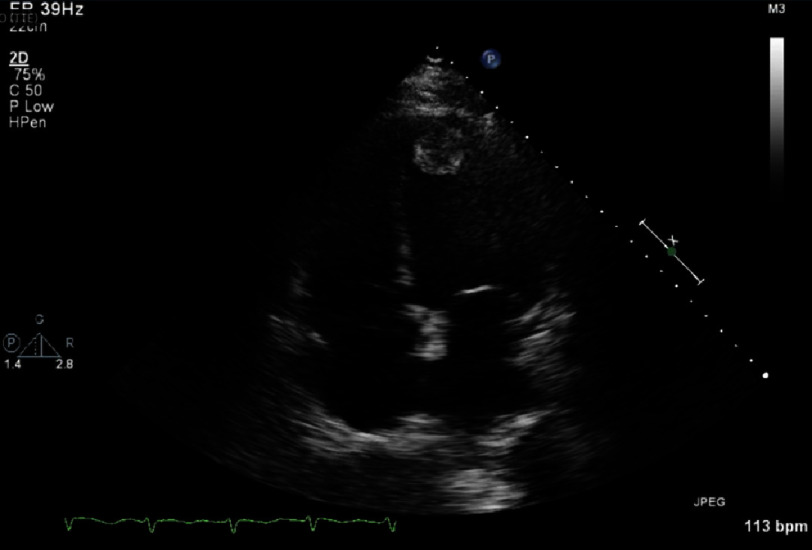
Transthoracic echocardiogram, apical four-chamber view demonstrating apical left ventricular thrombus measuring 24 ×27 mm.

Of note, transthoracic echocardiogram (TTE) showed severe left ventricle (LV) dysfunction with a left ventricle ejection fraction (LVEF) of 10–15% and evidence of an apical left ventricular thrombus 24 ×27 mm (Figure 1).

**Figure 2.**
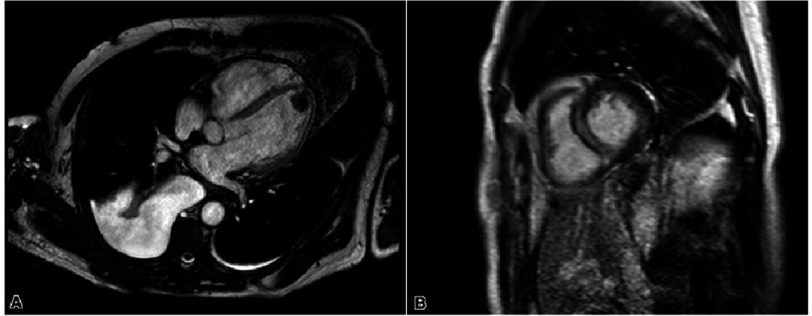
Four-chamber (A) and short-axis (B) cardiac magnetic resonance imaging demonstrating apical thrombus and focal delayed mid-wall myocardial enhancement at the basal portion of the interventricular septum consistent with myocarditis versus infiltrative process.

The patient was started on milrinone, bumetanide, amiodarone, and heparin infusion. Liver enzymes trended to the thousands range, for which amiodarone was stopped. He was then started on digoxin for rate control. Viral panel returned positive for human metapneumovirus, among other viruses tested. Subsequent cardiac magnetic resonance imaging (cMRI) showed EF 29% thrombus present and focal delayed mid-wall myocardial enhancement at the basal portion of the interventricular septum consistent with myocarditis versus infiltrative process (Figure 2).

Four days into the patient’s hospital course, he underwent right heart catheterization, which demonstrated normal bilateral filling pressures. A left heart catheterization revealed moderate to severe plaque in the mid-left anterior descending artery, moderate stenosis of the right posterior descending artery, and cardiomyopathy, which seemed to be out of proportion to the coronary artery disease. His weight was reduced by about 40 pounds following diuresis with improved kidney function, and he was weaned off of milrinone infusion accordingly. He was transitioned to oral diuretics and oral anticoagulation. The hospital course was complicated by expressive aphagia with an MRI brain showed multiple acute focal infarcts concerning for embolic etiology. Hemodynamics were improved towards the end of his hospitalization course, and he was discharged on day 12.

### Timeline

**Table:** 

Two months prior to admission	He experienced upper respiratory tract infection with progressive dyspnea on exertion.
Day 0	Admitted to the intensive care unit for severe hypoxia and severe volume overload.
Day 1	Started on intravenous bumetanide infusion with significant urine output. Rhythm was flutter on admission, started on intravenous amiodarone and heparin. TTE showed EF 10–15% and large (24 x 27 mm) spherical thrombus in the left ventricular apex. Liver enzymes jumped into the 4000s range, intravenous amiodarone stopped. He was started on intravenous milrinone.
Day 2	Transitioned to mg intravenous bumetanide twice daily. Milrinone and heparin continued. Digoxin was added for atrial flutter control. Liver enzymes improved.
Day 3	cMRI showed apical thrombus and findings concerning for myocarditis versus infiltrative disease and EF 29%.
Day 4	Cardiac catheterization showed normal intracardiac pressures with a CO/CI of 7.7 L/min and 4.2 L/min/m^2^ , wedge pressure 5 mmHg, pulmonary artery mean of 11 mmHg, and right atrial pressure 0.5 mmHg after the A wave. Milrinone weaned off. Further diuresis held. Tested positive for hMPV
Day 5	Carvedilol 3.125 mg and valsartan 40 mg twice daily were started and transitioned to oral furosemide 40 mg daily.
Day 7	Hospital course complicated by expressive aphagia. MRI brain showed multiple acute focal infarcts concerning for embolic etiology. Spironolactone 25 mg and dapagliflozin 10 mg were added.
Day 12	He remained hemodynamically stable with improvement of initial symptoms and was discharged accordingly.
Day 16	Patient was followed in the clinic. Carvedilol was increased to 12.5 mg twice daily.

## Patient 2

A 58-year-old female presented to the emergency department in May with progressively worsening dyspnea on exertion associated with upper respiratory tract infection three days before arrival. She has a history of breast cancer status post mastectomy and chemotherapy eight years ago and is currently on tamoxifen and hypertension.

On physical examination, the patient was found to be in profound respiratory distress, appeared to be cool and clammy, and lung auscultation with bilateral basal crackles with concerns of underlying cardiogenic shock. She had a temperature of 97.7 F, blood pressure of 117/92 mm Hg, heart rate of 114 beats per minute, and respiratory rate of 40 breaths per minute. Blood work revealed a white cell count of 9.2 B/L, hemoglobin of 12.2 g/dL, AST 67 IU/L, ALT 19 IU/L, lactate of 2.2 mmol/L, high sensitivity troponin T 1076 ng/L, and NT proBNP 1288 pg/mL. An EKG showed sinus tachycardia. Computerized tomography with angiography was negative for pulmonary embolism but showed patchy ground glass opacities bilaterally.

**Figure 3.**
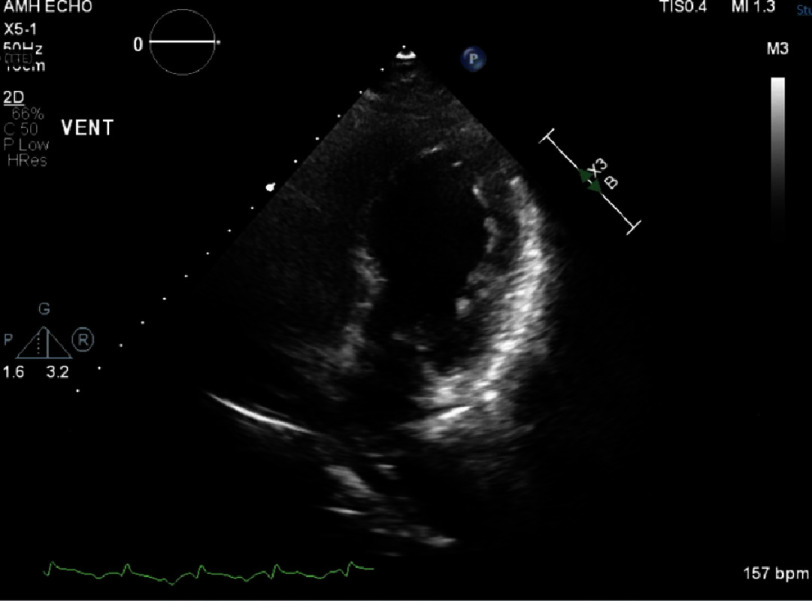
Transthoracic echocardiogram with two chamber views demonstrates apical akinesis and ballooning of the left ventricle.

She was initially managed with BiPAP but ultimately was intubated for hypoxia and continued respiratory distress. A stat TTE revealed severely depressed left ventricular systolic function with a left ventricle ejection fraction (LVEF) of 15–20% with apical akinesis and ballooning of the left ventricle thought to be stress-induced cardiomyopathy (Figure 3).

She was started on norepinephrine and was emergently brought to the catheterization lab with coronary angiography, which showed no evidence of obstructive coronary artery disease. A right heart catheterization revealed a severely depressed cardiac index with elevated filling. An Impella CP 2.5 device was placed, and the patient was moved to the intensive care unit. She was initially managed with intravenous diuresis with an appropriate response. Her hemodynamics improved with decreased vasopressor requirements, and she remained sedated and intubated. Differentials included stress-induced cardiomyopathy, viral myocarditis, and chemotherapy-related cardiotoxicity. The viral panel was negative for COVID-19, influenza A/B, EBV IgM/IgG, and parvovirus IgM/IgG but tested positive for human metapneumovirus. Notably, The patient had surveillance echocardiography three years before presentation and had normal LVEF.

She was extubated on the third day, and Impella was decannulated on day 5. Post decannulation, TTE showed improvement of LVEF to 30–35%. Cardiac magnetic resonant imaging (CMR) showed an LVEF of 59% and normal wall motion in the right and left ventricles. No definite delayed myocardial enhancement is seen to suggest a scar, infarct, or infiltrative disorder. High sensitivity troponin T level on day 3 was 282 ng/L. Given down-trending troponins and clinical improvements, endomyocardial biopsy was deferred. The patient remained clinically towards the end of her hospitalization course and was discharged on day 9.

The patient was clinically well on a follow-up visit seven days post-hospital discharge, although with a persistent cough thought to be related to post-viral syndrome.

### Timeline

**Table:** 

Three days prior to presentation	She experienced upper respiratory tract infection with progressive dyspnea on exertion.
Day 0	Intubated in emergency department and was started on norepinephrine for presumed cardiogenic shock. Impella CP was placed. Admitted to shock service for further workup. TTE: EF 15–20%, and ballooning of LV apex, normal RV.
Day 2	Extubated. Respiratory pathogens panel positive for hMPV
Day 4	Impella CP was decannulated with repeat TTE showing LVEF 30–35%.
Day 6	She was started on metoprolol succinate 12.5 mg daily.
Day 8	cMRI showed marked improvement of LVEF. She was started on spironolactone 25mg daily. Stopped coumadin and heparin. Cordis removed.
Day 9	She was clinically stable and was discharged home.
Day 16	She followed up in the clinic and was clinically stable.

## Discussion

hMPV, a respiratory pathogen recently identified within the Metapneumovirus genus of the Paramyxoviridae family, is distributed globally and exhibits seasonal variation, peaking in late winter and early spring. In Korea, as reported in our patients who both presented in May, hMPV co-circulates with influenza B from late March to early May. While hMPV is most prevalent in the pediatric population, advances in diagnostic technology have increased recognition as a significant pathogen in older adults. There has been emerging evidence that hMPV may result in extrapulmonary manifestations with possible cardiac involvement and severe complications.

A retrospective single-center study by Jubair et al. examined the hMPV association with cardiac morbidity involving 56 hospitalized patients aged 22-92 years with viral respiratory infections and no new cardiac events in the preceding three months. They were tested for hMPV, rhinovirus, and influenza virus through nasal swab samples. Among the 28 patients who tested positive for hMPV, a 32% incidence of cardiac events was observed despite the absence of recent cardiac morbidity. This represented a ten-fold increase in cardiac incidents compared to the control group, which tested positive for rhinovirus or influenza. Cardiac events, including new ischemic events, new arrhythmias, and myocarditis, were predominantly observed in the elderly, with 75% of affected patients being over 60 years old. The study concludes that hMPV significantly contributes to increased cardiac morbidity in older adults, even in the absence of other risk factors^4^. Similar to Case 1, the patient had no significant past medical history, although he succumbed to hMPV myocarditis and luckily experienced improvement in his condition.

Past reports indicate that myocarditis presenting in patients with new-onset heart failure and an influenza-like illness testing positive for a single viral pathogen only is typically shown to be viral myocarditis^5–7^. The diagnosis of viral myocarditis in both of our patients was supported by clinical history, lab findings, echocardiogram with severely reduced ejection fraction, and a cardiac MRI suggestive of myocarditis in one patient but not the other. CMRI often shows signs typical of myocarditis and is becoming routine in diagnosis^8^. The diagnosis on CMRI is based on the detection of edema on T2 weighted images and patchy or diffuse enhancement in mid-wall and/or subepicardial areas of the left ventricle^9^. While this enhancement pattern may be seen in other idiopathic and infiltrative processes, the acuity of clinical presentation and correlation with different clinical features of myocarditis differentiate myocarditis from these conditions. In addition, fibrosis on cardiac MRI has prognostic significance^10^. Dilatation of left heart chambers is frequently seen in acute myocarditis and indicates a worse prognosis^11,12^. We did not perform an endomyocardial biopsy as our patients improved significantly throughout hospitalization, and since it would not have changed management. Although a definitive diagnosis of viral myocarditis requires endomyocardial biopsy, its utility has been questioned as there are no specific indications for this procedure^13,14^.

Our patients presented with severe systolic dysfunction with variable duration of preceding symptoms of URTI who tested positive only for hMPV consistent with viral myocarditis. They were treated with pressor/inotrope support as indicated and were initiated on goal-directed medical treatment for heart failure as tolerated. They demonstrated rapid improvement in their condition and were closely followed by a multidisciplinary team. Other reported cases of adult hMPV myocarditis are described in Table 1.

**Table 1:** Overview of reported cases of adult hMPV myocarditis in the literature.

**Author**	**Reference**	**Age/Sex**	**Symptoms**	**Comorbid conditions**	**Echocardiogram findings**	**CMR findings**	**EMB**	**Treatment**	**Length of stay**	**Follow up**
Weinreich et al.	^15^	25/M	URTI	None	LVEF 25%, severely dilated LA and LV.	Septal wall enhancement	None	IV diuretics, ACEinh, BB	7 days	Not reported
Gopalan et al.	^16^	27/M	URTI, tachycardia, & lower extremity edema	CKD-III, nephrotic syndrome, pleural effusions, and T2DM	Initial: LVEF 54% and apical pericardial effusion. 2 month follow up TTE: LVEF 37%	Pericardial and bilateral pleural effusions, diffuse myocardial edema	None	Not reported	Not reported	Not reported
Bhatia et al.	^17^	53/M	URTI & SOB	None	Initial: LVEF 35–40% with global hypokinesia and mild pericardial effusion. 3 days: LVEF 50–55%	Not reported	None	IV diuretics, ACEinh, BB, nitrates	6 days	Not reproted
Choi et al.	^18^	73/F	URTI & dyspnea	HTN, DM and Afib	LVEF 50–55%, small pericardial effusion	Not reported	None	Supportive management	7 days	Not reported

**Notes.**

URTI Upper respiratory tract infection SOB Shortness of breath LVEF Left ventricular ejection fraction IV intravenous HTN Hypertension DM Diabetes mellitus Afib Atrial fibrillation

### What have we learned?

• Consider hMPV as a potential etiological agent in myocarditis, especially in older adults presenting with concurrent respiratory and cardiac symptoms.
• Recognize that hMPV may be associated with fulminant myocarditis in patients with no significant history of immunosuppression or underlying cardiac disease.

## References

[1] Van den Hoogen BG, de Jong JC, Groen J, Kuiken T, de Groot R, Fouchier RAM, Osterhaus ADME ((2001)). A newly discovered human pneumovirus isolated from young children with respiratory tract disease. Nature Medicine.

[2] Haas LE, Thijsen SF, van Elden L, Heemstra KA (2013). Human metapneumovirus in adults. Viruses.

[3] Panda S, Mohakud NK, Pena L, Kumar S (2014). Human metapneumovirus: review of an important respiratory pathogen. International Journal of Infectious Diseases.

[4] Jubair W, Grigg S, Junqueira MJ (2020). Human metapnemovirus infections are associated with increased cardiac morbidity. Elderly Patients.

[5] Lobo MLS, Taguchi Â, Gaspar HA, Ferranti JF, de Carvalho WB, Delgado AF (2014). Fulminant myocarditis associated with the H1N1 influenza virus: case report and literature review. Revista Brasileira de Terapia Intensiva.

[6] Ohara N, Kaneko M, Kuwano H ((2015)). International Heart Journal.

[7] Flor de Lima B, Silva J, Rodrigues AC, Grilo A, Riso N, Riscado MV (2013). Hand, foot, and mouth syndrome in an immunocompetent adult: a case report. BMC Research Notes.

[8] Hundley WG, Bluemke DA, Finn JP (2010). ACCF/ACR/AHA/NASCI/SCMR 2010 expert consensus document on cardiovascular magnetic resonance: a report of the American College of Cardiology Foundation Task Force on Expert Consensus Documents. Circulation.

[9] Goitein O, Matetzky S, Beinart R, Di Segni E, Hod H, Bentancur A, Konen E (2009). Acute myocarditis: noninvasive evaluation with cardiac MRI and transthoracic echocardiography. American Journal of Roentgenology.

[10] Moraes GL, Higgins CB, Ordovas KG (2013). Delayed enhancement magnetic resonance imaging in nonischemic myocardial disease. Journal of Thoracic Imaging.

[11] Felker GM, Boehmer JP, Hruban RH, Hutchins GM, Kasper EK, Baughman KL, Hare JM (2000). Echocardiographic findings in fulminant and acute myocarditis. Journal of the American College of Cardiology.

[12] Anzini M, Merlo M, Sabbadini G, Barbati G, Finocchiaro G, Pinamonti B, Salvi A, Perkan A, Di Lenarda A, Bussani R, Bartunek J, Sinagra G (2013). Long-term evolution and prognostic stratification of biopsy-proven active myocarditis. Circulation.

[13] Das BB (2014). Role of endomyocardial biopsy for children presenting with acute systolic heart failure. Pediatric Cardiology.

[14] Cooper LT, Baughman KL, Feldman AM, Frustaci A, Jessup M, Kuhl U, Levine GN, Narula J, Starling RC, Towbin J, Virmani R (2007). The role of endomyocardial biopsy in the management of cardiovascular disease: a scientific statement from the American Heart Association, the American College of Cardiology, and the European Society of Cardiology. Circulation.

[15] Weinreich MA, Jabbar AY, Malguria N, Haley RW (2015). New-Onset Myocarditis in an Immunocompetent Adult with Acute Metapneumovirus Infection. Case Rep Med.

[16] Gopalan R, Vargas Rodriguez A, Hopkins M, Andrade A, Ung R, Kayla A, Silver M (2024). Metapneumovirus: A Rare Agent Causing Myocarditis. Journal of Cardiac Failure.

[17] Bhatia A, Joshi S, Tayal N (2023). Rare myocarditis following acute metapneumovirus infection. Indian Journal of Critical Care Case Report.

[18] Choi MJ, Song JY, Yang TU, Jeon JH, Noh JY, Hong KW, Cheong HJ, Kim WJ (2016). Acute Myopericarditis caused by Human Metapneumovirus. Infection & chemotherapy.

